# 25 years of African trypanosome research: From description to molecular dissection and new drug discovery^☆^^☆☆^

**DOI:** 10.1016/j.molbiopara.2015.01.006

**Published:** 2015

**Authors:** Keith R. Matthews

**Affiliations:** Centre for Immunity, Infection and Evolution, Institute for Immunology and Infection Research, School of Biological Sciences, University of Edinburgh, Edinburgh EH9 3JT, UK

**Keywords:** *Trypanosoma brucei*, Molecular parasitology, Trypanosome

## Abstract

The Molecular Parasitology conference was first held at the Marine Biological laboratory, Woods Hole, USA 25 years ago. Since that first meeting, the conference has evolved and expanded but has remained the showcase for the latest research developments in molecular parasitology. In this perspective, I reflect on the scientific discoveries focussed on African trypanosomes (*Trypanosoma brucei* spp.) that have occurred since the inaugural MPM meeting and discuss the current and future status of research on these parasites.

## Highlights

• The Woods Hole ‘Molecular Parasitology Meeting’ celebrated its 25th meeting in 2014.
• This ‘Perspective’ overviews discoveries in trypanosome biology since 1990.
• New tools for gene function discovery have transformed trypanosome research.
• Trypanosomes remain fascinating parasites with a unique biology.
• This offers the potential to provide new therapeutic options.

## 1 Introduction

**Fig. 1:**
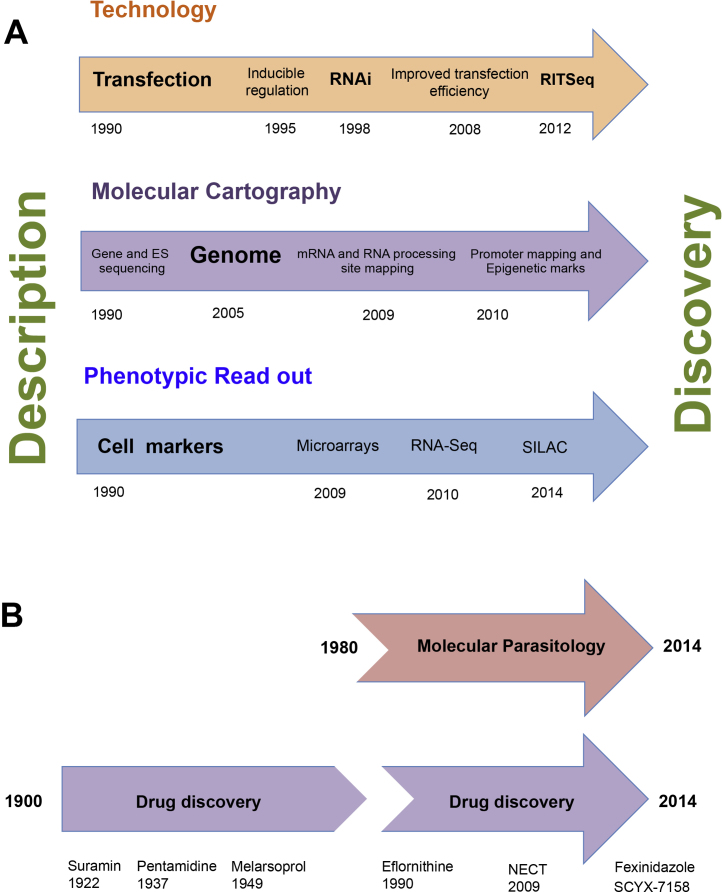
(A) Progress in trypanosome biology over the last 25 years. Developments in the understanding of trypanosome biology have been driven by the development of new technologies (“Technology”), the availability of new datasets (“Molecular Cartography”) and through the utility of cytological and molecular markers or expression profiles (“Phenotypic read out”) that have assisted the interpretation of genetic perturbations. These developments have progressed the field from an era of description to one where gene function can be discovered and understood. B. Timescales of new drug discovery for African trypanosomiasis and molecular parasitological research. The major drugs for Human African Tryapnosomiasis are old, and there is an important need for new drugs. The development of molecular parasitology as a field promises to accelerate new drug discovery through the identification of important processes and targets in the parasite. However, the discovery and development of new drugs is slow and expensive such that the impressive discoveries that have emerged from molecular parasitology are only now beginning to yield new potential new therapies. This has been driven by an increasing focus and resource investment into the search for new drug targets as a complement to new biological understanding *per se*.

The Annual Molecular Parasitology Meeting started in Woods Hole on September 9th 1990 and, with the exception of the tragically disrupted conference in September 2001 (when a mini-meeting was held involving those able to attend), has hosted 300 or more delegates for 25 years. Woods Hole was the natural home for the conference, it being the base of the long running Biology of Parasitism Summer School, and has provided many young molecular parasitologists with their first exposure to a vibrant, sometimes intimidating, international research conference [1]. Although the emphasis of the conference has changed over the years, such that the early presence of (even then only a few) helminth molecular biologists diminished and apicomplexan biology has come to dominate, a core focus on trypanosomes and particularly African trypanosomes has remained. In this perspective, I reflect on the breath-taking developments in our knowledge of the biology and molecular biology of *Trypanosoma brucei* that have occurred over the last 25 years and highlight the discoveries, technologies and controversies that have made this conference such an exciting event in our scientific calendar (Fig. 1A). I also consider, almost certainly naively, how research into trypanosomes may develop in future. I emphasise also that this perspective is personal and not comprehensive; I have certainly omitted many key discoveries and debates either through poor recollection or through simply missing key sessions whilst in the Captain Kidd. Notably, I have focussed on the developments in the molecular and cellular biology of trypanosome parasites to the detriment of many of the biochemical processes that have proved so fascinating over the last 25 years.

## 2 The genome

Although not formally published as a draft genome until 2005 [2], sequencing efforts underpinned many of the discoveries in trypanosome biology far earlier. In the late 1980s the state of the art represented chromosome walking along VSG gene transcription units through the isolation of overlapping bacteriophage lambda clones, allowing a description of the architecture and gene composition of the telomeric expression sites [3–5]. Beyond this, gene sequence analysis involved cloning from plasmid and phage libraries after hybridisation with labelled cDNA or oligonucleotide probes and, from the late 1980s, exploitation of the recently-discovered PCR technology using (at the time expensive) oligonucleotide primers. Systematic genome sequencing efforts began with Chromosome Ia (funded initially by the WHO) and thereafter Chromosomes IX, X, XI and Chromosomes II-VIII, funded by the Wellcome Trust and NIAID respectively, with leadership from (among others) Sara Melville, Andy Tait and Keith Gull (in the UK) and Elisabetta Ullu, Ken Stuart, George Cross and John Donelson (in the US). These efforts focussed on *T. brucei* TREU 927/4, a strain competent for progression through the entire life cycle [6], though most lab research at the time and subsequently has focussed on *T. brucei* Lister 427, which compromises some biological characteristics (pleomorphism, efficient tsetse passage) for rapid growth *in vivo* and *in vitro* and a low frequency of antigen switching, this facilitating some studies on antigenic variation. Since the publication of the genome for *T. brucei* (coincidently with *Trypanosoma cruzi* and *Leishmania major*), assemblies of several further trypanosome genomes have been generated, including different strains and species (*T. congolense*, *T. vivax*, *T. evansi*, *T. grayi*; http://tritrypdb.org) whereas the difficult to clone and assemble telomeric sequences have been derived through TAR cloning, allowing expression site architectures to be compared across the genome and between species [7,8]. All of these efforts, of course, have made an enormous contribution to our ability to interrogate gene function, gene expression and genome regulation and almost every publication today in trypanosome molecular parasitology makes use in some way of the information generated, and made accessible through the efforts of the genome sequencers, curators and bioinformaticians.

## 3 Antigenic variation

Trypanosome populations escape immune clearance by antibody responses through their capacity for antigenic variation. The protein responsible, the variant surface glycoprotein, was well characterised in 1990, and the structure solved to 2.9-angstrom resolution [9]. The N- and C-terminal domains provide for antigenic diversity and membrane association, respectively, with antibodies being inaccessible to the more conserved membrane-proximal domain on live parasites [10]. The protein is anchored to the lipid membrane *via* the glycosylphosphatidylinositol (GPI) lipid anchor and the pathway of its assembly was established 25 years ago [11–13]. However, since then characterisation of the enzymes involved in the synthesis and addition of this essential component of the surface coat of the parasite has provided a detailed description of its assembly pathway, these steps also providing potentially excellent targets for drug development efforts [14–16]. The fluidity of the VSG on the parasite surface enabled by GPI anchoring and its rapid recycling by the parasite [17] also revealed a new facet of the VSG's contribution to immune evasion, namely hydrodynamic flow [18]. Here, bound antibodies are swept towards the flagellar pocket of the parasite for endocytosis through the swimming action of the parasite in blood. Although this only delays the inevitable destruction of the parasite as antibody titres increase, the increased survival time of the parasite might provide a useful component of their infection dynamics immediately prior to clearance of a given variant type by the immune system. Hydrodynamic flow is particularly effective on transmission stage stumpy forms of the parasite, and its action might therefore prolong the lifespan of these irreversibly arrested parasites, increasing their probability of tsetse uptake.

In the late 1980s, a combination of run-on transcription analysis and analysis of the sensitivity of transcription units to UV-induced nucleotide dimerization demonstrated that VSG expression sites were polycistronic [3,5,19,20], with a distant upstream promoter driving transcription through a number of expression site associated genes (ESAGs) before reaching the telomere-proximal VSG gene itself. The polymerase responsible for transcription was known to be unusual, with RNA polymerase II (polII) lacking the conventional C terminal extension [21], and inhibition studies using the RNA polII inhibitor alpha amanitin having indicated that VSG expression site transcription was mediated by RNA polymerase I (polI) [22–24]. Formal biochemical support for this using *in vitro* transcription assays and polymerase subunit depletion was provided in 2003 from the Gunzl lab [25], with the unusual nature of the polI complex being subsequently revealed by affinity purification [26].

The mechanism of antigen switching was also well characterised before 1990, with a classical cassette-based model for VSG switching, whereby intact antigen encoding genes were activated either by changes in the activity of an expression site (from around 15 to 20) or from translocation into an active expression site by gene conversion or reciprocal telomeric exchange [27,28]. With a large repertoire of VSG genes available in the trypanosome genome (>1000 based on hybridisation studies; [29]) this provided a significant pool of available antigen types to ensure long-term immune evasion. Although this model provided a textbook mechanistic basis for the gene rearrangements associated with antigen switching in trypanosomes, analysis of the genome revealed that it was an oversimplification. Rather than a pool of intact VSG genes, the genome was found to contain a majority of incomplete VSG genes that required to be assembled into functional mosaics [30,31]. This was a surprise, with immediate implications for the potential size of the available VSG repertoire (essentially infinite) and also the probability of VSG gene activation and the ordering of the appearance of new antigen types [32].

The existence of several expression sites raised the question of how exclusive activation of only one expression site at a time was achieved, and also why more than one expression site was necessary. The first of these questions was resolved by the discovery of an extranucleolar RNA polymerase I transcription factory, termed the expression site body (ESB) [33]. This provided a positional and structural entity where monoallelic exclusion could be achieved through the restricted association of only one telomeric expression site to the ESB at any one time, with control being assisted by epigenetic mechanisms. Recently regulators of the telomere silencing machinery have been uncovered, bringing molecular understanding of the long-recognised stringent control of ES repression [34–37]. Although both VSG and procyclins, the surface proteins expressed on the procyclic stage of the parasite, are both polI transcribed, the ESB was bloodstream form specific, with telomeric association with the nuclear periphery being lost during differentiation from the bloodstream to the procyclic forms. In procyclic forms procyclin transcription is nucleolar [38].

A potential answer to the second question, a reason for the existence of more than one expression site, was suggested after the discovery that the promoter-proximal expression site associated genes, ESAG6 and 7, encoded a heterodimeric receptor, responsible for the uptake of iron from the host bloodstream in the form of transferrin [39]. The existence of microheterogeneity in the ESAG6 and 7 sequences from different expression sites led to the hypothesis that different expression sites might be optimised for transferrin uptake in different hosts [40], which exhibit subtly different transferrins. This attractive model proposed that the activity of different ES would provide a growth advantage in different host sera, but experimental support remains equivocal [41]. A second expression site associated gene, ESAG4, was known to encode an adenylate cyclase activity [5,42] although its function was unclear until recently. Studies focussed on the early infection of parasites expressing a dominant negative form of ESAG4 (necessary because the large number of ESAG4 genes in the genome precluded either gene knockout or gene silencing approaches) revealed that parasites were less able to establish infection in a mouse model [43]. This was proposed to be due to the effect of the ESAG4 activity on macrophages when trypanosomes were phagocytosed, the consequence of which was to inhibit macrophage activation and so promote the survival of parasites still circulating in the bloodstream. Other expression site associated genes remain of cryptic function although three (ESAGs 1, 2 and 8) have been reported to be involved in the developmental capacity of parasites undergoing VSG switching [44], with one of these (ESAG8), proposed to be nucleolar, also being potentially involved in translational regulation through the action of Puf proteins [45,46].

## 4 Human serum resistance and trypanolytic factors

Although the function of many ESAGs remains unclear, one ESAG, found only in *T. brucei rhodesiense*, has a confirmed role in the resistance of these parasites to human serum trypanolytic factors. Raymond Hamers identified mRNAs associated with parasites that were resistant to human serum lysis [47,48] and after involvement with Patrick De Baetselier, Etienne Pays demonstrated the gene responsible for conferring resistance was an expression site associated gene apparently derived from a VSG sequence, termed serum resistance associated (SRA) [49]. SRA was found in a truncated expression site and its expression was directly linked to serum resistance, both in parasites that fluctuated in their serum resistance phenotype (due to different expression site usage, where only one expression site contained SRA) and in transgenic *T. brucei* engineered to express SRA. Indeed, SRA was found to be diagnostic for *T. b rhodesiense* providing a key field tool to identify human infective parasites circulating in a zoonotic infection cycle [50,51].

The mechanism of SRA action and the trypanolytic components of human serum have been controversial over the last 25 years, but a consensus has emerged. Human serum contains trypanolytic activities within high density lipoprotein (TLF1) and IgM complexes (TLF2). TLF1, which contains haptoglobin-related protein (HPR) in complex with haemoglobin, is taken up by trypanosomes through their haptoglobin/haemoglobin receptor (TbHpHbR) [52]. TLF2, in contrast, does not enter the trypanosome *via* the TbHpHbR but instead seems to be internalised after relatively non specific interaction, possibly with the VSG at the parasite surface. This pathway is more relevant at physiological concentrations of HPR, where haptoglobin concentrations render the TLF1 pathway likely inoperative through receptor competition [53,54]. Both TLF1 and TLF2 mediate trypanosome killing through the action of ApoL1 [55,56], which inserts in the endosomal membrane and, upon entering the lysosome permeabilizes the membrane causing swelling through chloride ion influx and then osmotic lysis. Resistance in *T. b. rhodesiense* is mediated through SRA binding to ApoL1 during trafficking to the lysosome, which inactivates its lysosomal pore forming capabilities [57,58].

The SRA gene is absent in *T. b. gambiense*, where human serum resistance is mediated by an alternative protein also apparently derived from a VSG sequence [59,60]. This molecule, TgsGP, acts to rigidify the lysosomal membrane, rendering it less susceptible to permeabilization by ApoL1, thereby preventing parasite lysis [60]. Uptake of TLF1 is also limited in *T. b gambiense* parasites by inactivation of the TbHpHbR, which prevents parasite killing under conditions of hypohaptoglobinaemia, common in malaria endemic regions through selection for the sickle cell trait. Receptor inactivation was necessary because affected individuals release haemoglobin reducing the abundance of haemoglobin-haptoglobin complexes [61], favouring the TLF1 pathway.

As well as parasite adaptive mechanisms, trypanosome hosts have also evolved effective countermeasures to protect themselves from parasite infection. The evolution of APOL1 in old world monkeys conferred resistance to *Trypanosoma brucei* spp., whereas in baboons the ApoL1 C-terminal sequence enables trypanolytic activity even in the presence of SRA [62]. This represents an interesting potential response to the evolution of trypanosomes able to escape ApoL1 mediated lysis, a phenomenon also detected in human populations where two mutations in ApoL1 are enriched in populations of recent African origin, albeit with a concomitantly increased risk of kidney disease [63,64].

## 5 Cell structure, cell cycle and life cycle

The first quantitative temporal descriptions of the trypanosome cell cycle were published in 1989 [65], with the kinetoplast and nuclear replication and division cycles being described in 1990 [66]. These have provided the template for many analyses of phenotypes generated upon gene depletion by RNAi and gene knock out. For the complex network of kinetoplast DNA, minicircle replication has been found to involve detachment from the compacted kDNA network, replication at antipodal replication sites, followed by reinsertion into the network [67,68]. The kDNA is itself segregated through attachment to the basal body *via* a filamentous network, the tripartite attachment complex [69], that spans from the kinetoplast DNA, through the mitochondrial membrane, attaching ultimately to the basal body of the trypanosome flagellum. The molecular components of this machinery have started to be identified [70–72], with one component, AEP1, apparently being generated by the alternative RNA editing of COXIII transcripts [73]. Failure to successfully replicate or segregate the kDNA results in the generation of dyskinetoplastid (DK) parasites, lacking a mitochondrial genome. Although these are inviable in the insect vector, DK parasites can survive in the bloodstream in the presence of a mutation in the F_1_F_O_ ATPase gamma subunit that allows them to generate a mitochondrial membrane potential in the absence of kDNA-encoded factors [74,75].

For nuclear DNA, early pulsed field gel electrophoresis studies of the chromosome events associated with antigenic variation had revealed the existence of large numbers of minichromosomes, as well as the 11 diploid chromosomes [76,77]. The minichromosomes apparently increase the pool of telomeric VSG genes for antigenic variation, but necessitate a distinct spindle apparatus to that of higher eukaryotes to allow chromosome segregation in the absence of a kinetochore and microtubule structure for each and every chromosome. This is achieved through microtubules that run pole–pole, allowing 50–150 kB minichromosomes to be segregated with fidelity [78]. For the megabase chromosomes, the site of kinetochore binding at centromeres was unclear until a recent iterative immunoprecipitation process allowed the isolation of a coherent kinetochore protein complex unlike that identified in any eukaryote to date [79]. The replication machinery for nuclear DNA is also unusual, particularly in the formation of the early replication complex. Nonetheless, the components are beginning to be elucidated and this reveals features in common with other eukaryotes, including the presence of multiple, albeit widely spaced, origins perhaps to avoid interference with the transcription machinery operating on the long polycistronic transcription units that characterise gene organisation in kinetoplastid parasites [80,81].

As well as the as DNA containing organelles, there has been considerable focus over the last 25 years on the structure of the other single copy organelles within the trypanosome cell. A particular focus has been on the flagellum, where the genetic tractability of trypanosomes has provided an excellent model for eukaryotic flagellar biology, an area of significant and widespread interest through the role of flagellar and cilia defects in several human diseases [82–84]. The flagellum comprises the conventional 9 + 2 axoneme plus an associated paraflagellar rod structure that assists parasite swimming [85]. In the procyclic forms of the parasite, positional information for flagellar duplication is provided by a flagellar connector structure that ensures that the new flagellum tracks along the path of the old flagellum [86] with a subpellicular flagellum attachment zone zippering the flagellum along the cell body. Whilst fundamental, the absence of an easily recognisable flagellum connector in bloodstream form parasites [87] remains a conundrum. Protein trafficking along the flagellum during its growth and once established is achieved *via* intraflagellar trafficking similar to the process seen in other eukaryotes [82]. This is accompanied during outgrowth of a daughter flagellum by detailed positional restructuring as the flagellum emerges from the parasite's flagellar pocket, a precisely organised invagination of the cell surface that controls membrane recycling and exchange with the environment [88]. This establishes the correct cellular architecture necessary for the production of a viable daughter cell [89].

The same problem accompanies the replication and segregation of other single copy organelles. The Golgi, for example, is segregated in association with a bilobe structure [90,91] located close to the mouth of the flagellar pocket and aligned with the flagellar attachment zone. This complex is replicated with fidelity in procyclic forms, but less so in bloodstream forms. The secretory path is also similar to that of other eukaryotes but tuned for efficient VSG trafficking in the bloodstream forms [92]. Indeed, endocytosis is far more active in bloodstream forms than procyclic forms, with Rab11 apparently being a key player in the process, acting in concert with other conserved and evolutionarily divergent components of the endocytic apparatus to enable VSG recycling and the removal of VSG associated immunoglobulins *via* the lysosome [93]. The mitochondrion also requires replication and segregation of its genome, as well as duplication and segregation of the organelle itself. Both processes depend on import of nuclearly encoded mitochondrial proteins. The import machinery is not well conserved with respect to other eukaryotic organisms and instead the basic outer membrane import protein was suggested to be of bacterial origin [94]. A further organelle type linking eukaryotic and prokarytic cellular evolution was the discovery of acidocalcisomes in *T. cruzi* by Roberto DoCampo [95]. These provide an acidic calcium store enriched in Pi, PPi and polyphosphate and have been found in other kinetoplastids, but also apicomplexans, *Chlamydomonas*, bacteria and also metazoa – suggesting their origins prior to the divergence of eukaryotic and prokaryotic cells [96]. These appear to have shared functions to control osmotic balance as well as cation and phosphate stores, with the discovery of IP_3_R in the acidocalcisomes of trypanosomes also indicating a role in cell signalling [97,98].

## 6 Life cycle

Life cycle events differ importantly between different African trypanosome species, but most understanding and progress has been made with *T. brucei*. In the 1980s almost all laboratory work focussed on laboratory-adapted bloodstream form lines, termed monomorphs, and long-term cultured procyclic forms. However, in 1990 Ziegelbauer and colleagues [99] described the synchronous differentiation of bloodstream to procyclic forms using pleomorphic parasites (*i.e.* those capable of generating transmissible stumpy forms, a trait reduced in laboratory-adapted monomorphic lines) stimulated by the differentiation triggers citrate and cis-aconitate. This enabled the tractable dissection, *in vitro*, of developmental events as parasites differentiated from arrested bloodstream stumpy forms, which accumulate at peak of parasitaemic waves in rodent infections, to procyclic forms. Early studies established that stumpy forms were uniformly arrested in their cell cycle in G1 [100,101] and underwent a simultaneous cell cycle re-entry and differentiation within a matter of hours *in vitro*, with differentiation competence proposed to be linked to cell cycle arrest in G1/G0 [101]. Soon after differentiation the parasites express different isoforms of the procyclic form surface coat, initially EP and GPEET procyclin (early procyclic forms) but then only EP procyclin (late procyclic forms) [102–104]. In the fly's salivary glands, the insect forms undergo further development to epimastigote forms after an asymmetric division, where they express a new surface protein BARP, this eventually being replaced by VSG upon differentiation to metacyclic forms [105]. The physiological stimuli for the various developmental events during the life cycle remain unclear in most cases, though the production of stumpy forms seems to be stimulated by a parasite-derived signal termed stumpy induction factor [106]. Whatever the specific molecular trigger, numerous components of the signalling response pathway required for slender-to-stumpy differentiation have recently been characterised [107]. Once generated, stumpy forms are able to perceive entry into the tsetse fly through their expression of the surface PAD family of proteins that convey the citrate/cis-aconitate signal [108], to which they are hypersensitive at low temperature [109]. Signalling upon entry into the tsetse fly is mediated by a phosphatase-signalling cascade that is trafficked to the glycosomes [110–112].

After differentiation from early to late procyclic forms, an event promoted by hypoxia and/or glycerol exposure *in vitro* [102], procyclic forms multiply until passage through to the salivary glands, where epimastigotes form after an asymmetric division [113,114]. The journey to the salivary glands represents a strong bottleneck in the life cycle of the parasite [115], after which epimastigotes multiply whilst attached to the salivary gland wall. Some of these cells then undergo meiosis and sexual exchange, these events having been visualised by the expression of conserved meiosis specific proteins [116] and the appearance of fused cells after dual infection of tsetse flies with parasites expressing either red or green marker proteins, generating yellow cells [117].

Finally metacyclic forms are produced, which are arrested in division and re-express the VSG in preparation for infection of new mammalian hosts. Recapitulating *in vitro* the generation of metacyclic forms in the salivary glands from epimastigote forms has recently been achieved for *T. brucei*, this being driven, albeit inefficiently, by the overexpression of a small RNA binding protein, RBP6 [118]. This highlights that single protein expression changes can drive surprisingly complex developmental events, an observation reinforced by the morphological events induced by perturbed expression of the ALBA 3 and 4 proteins [119] and a calpain-like protein [120] and gene regulatory changes linked to perturbing the RNA helicase DHH1[121]

## 7 Post-transcriptional gene expression regulation

The organisation of the trypanosome genome into polycistronic transcription units was established in the late 1980s, reflecting that it was not only the VSG gene transcription unit that contained multiple genes but that this was a general genome-wide phenomenon [122,123]. Despite this multi-gene organisation, there remains little evidence for co-regulated operons such that the emphasis of gene regulation in trypanosomes is post-transcriptional. Transcription is initiated at the boundary of, and in some cases within, monodirectional gene clusters from ill-defined polymerase II promoter sequences marked by an enrichment of the histone variants H4K10ac, H2AZ, H2BV, H3 V and H4 V and the bromodomain protein BDF3 [124]. The transcription complex and the structure of individual transcription factors is unusual [125–127], with the primary transcripts from polycistronic transcription units being co-transcriptionally processed by trans splicing at the 5′ end of each mRNA and polyadenylated at the 3′ end, in a mechanistically coupled process [128]. The paradigm that there is an absence of cis-splicing still holds true for almost all genes in the trypanosome genome, although two genes have been discovered that exhibit cis-splicing [129], and the importance of alternative splicing to create distinct protein products, or proteins with altered localisation signals is becoming apparent [130,131]. Transcription termination at the boundaries of polycistronic transcription units appears to be associated with an accumulation of the Base J [132], a novel nucleotide produced in trypanosomes by the action of two biosynthetic enzymes [133].

To achieve differential mRNA abundance for transcripts derived from the same transcription unit, differential mRNA stability seems to be the dominant mechanism of control. The most definitive early studies focused on the procyclin mRNAs, where the focus of regulation operates through the 3′ UTR (though transcriptional control also contributes to this RNA polI transcribed gene family) [134]. Both positive and negative elements were mapped using reporter assays, suggesting the existence of regulatory loops controlling mRNA stability/instability and translational efficiency. For GPEET procyclin, environmental factors were also able to regulate developmental expression, such that the 3′ UTR contained sequence motifs responsive to the presence of environmental glycerol or hypoxia [102]. The focus of gene regulation on the 3′ UTR of regulated genes has remained a common theme through the analysis of many differentially expressed genes and gene families, but the identification of conserved linear or obvious secondary structural motifs has remained challenging. This partly reflects the difficulties in accurately predicting folding profiles for mRNA sequences by computational means alone, but also is a likely consequence of the use of multiple combinations of different regulatory RNA binding proteins to achieve complex regulatory events for co-regulated gene products.

Consistent with the post-transcriptional focus of gene regulation in trypanosomes, the parasite genome encodes a large repertoire of potential RNA regulators, some with confirmed functions in the control of stage-regulated genes [135]. After 15 years effort, regulators of procyclin mRNAs have emerged [136,137], and more recently a regulatory factor controlling many membrane-associated proteins has been identified [138], as has a stem-loop structure present on purine responsive genes [139]. In addition to these specific regulatory factors and sequences, more general regulatory factors have been characterised in detail. Hence, the core machinery linked to mRNA decay has been characterised and conserved components of the translational apparatus have been described [140,141]. As well as these machineries, the molecular cartography of gene expression and protein expression changes associated with different tractable parasite life cycle stages has been achieved, starting with differential display and subtractive hybridisation approaches, and thereafter by genomic arrays and microarrays, SAGE and RNA-Seq [142] and, most recently, SILAC proteomics and phosphoproteomics [143,144], ribosome profiling [145] and description of the small proteome [146]. These have led to increasingly detailed descriptions of the molecular profile of trypanosome developmental forms such that published differences are now less due to differences in methodology and resolution and more due to biological differences (media, isolation procedures, strains, growth characteristics, *etc.*).

Whether specific genes are perturbed or not, these outputs all provide data resources that will hopefully allow interconnected regulatory events to be understood, clarifying how transcriptional, mRNA processing, mRNA turnover, translational and post translational regulatory events are integrated to generate a physiological response. This kind of integrated modelling at the biochemical level was pioneered in the trypanosome system through the genetic and biochemical analysis of glycolytic compartmentation in specialised peroxisomes, the glycosomes [147–149]. This revealed that compartmentation of the glycolytic enzymes within the glycosomes was a key parameter to prevent glycolysis running out of control through the turbo design of the process, given the lack of feedback control mechanisms typical for other eukaryotes. This iterative interaction between mathematical prediction and experimental observation provided one of the earliest examples of a systems approach to understand biological networks [150], and has considerable potential to identify vulnerable biochemical control points to target pharmacologically. Many of these have been identified over the last 25 years, highlighting that trypanosomes exhibit extreme biochemical diversity as well as molecular and cellular diversity between life cycle stages and between different trypanosome species [151,152]. Highlighting these distinctions, bloodstream forms of the parasite use acetate produced in the mitochondrion *via* an ‘acetate shuttle’ and threonine to generate fatty acids, a process which is lethal if inhibited [153]. Similarly lipid biosynthesis in trypanosomes differs from mammalian hosts, offering targets for chemotherapy [154,155].

As well as nuclear gene expression, mitochondrial gene expression has remained an area of intense research activity. In 1990 the phenomenon of RNA editing was well established, with the role of the guide RNAs as templates of the process, and minicircles as the source of gRNA genes being reported in that year [156,157]. The mechanistic details of the process have been filled in considerably since then, with components of the editing machinery (the editosome) being identified, and uridine insertion and deletion activities characterised [158]. When combined with the RNA components, the 20S editosome protein complex – which contains the reaction centre that achieves mRNA cleavage, U insertion or deletion and religation – has been proposed to form a 35–40S complex [158]. Whilst this description of the components and action of the different subunits of the editosome has advanced significantly, providing potentially important drug targets [159], it emerged that editing is but one component of a complex mitochondrial gene expression network, with pentatricopeptide repeat-containing proteins assuming a key role [160]. A clear hypothesis for the function of the energetically expensive editing process remains elusive, although intriguing possibilities for how and why it might have evolved have been proposed [161,162].

## 8 Genetic tools to understand gene function

The rapid experimental advances over the last 25 years detailed above have been enabled by a series of technical breakthroughs such that trypanosomes now represent one of the most tractable microbial eukaryotes. In 1990, transient transfection of trypanosomes was possible [163,164] and the first MPM meeting reported homologous recombination of constructs into the trypanosome genome allowing the selection of the stable transgenic parasite lines needed for biochemical and molecular analysis. This was a key breakthrough since prior to that point the sophisticated molecular analyses of trypanosomes were necessarily descriptive. However, with gene knockout feasible through efficient homologous recombination, the ability to create null mutants, or to ectopically overexpress genes allowed experimental intervention and phenotypic analysis to uncover novel biology. Key to these early analyses was the development in the Clayton and Cross laboratories of inducible expression systems based on the incorporation of tetracycline operator sequences into the identified procyclin gene promoter, and subsequently T7 RNA polymerase promoter, such that expression could be regulated by tetracycline in a cell line expressing the tetracycline repressor protein [165–167]. Although this allowed the development of conditional knockout out approaches to analyse gene function, the diploid genome of the parasite in both bloodstream and procyclic forms, and the relative inefficiency of stable transfection (particularly in the bloodstream form) meant that the analysis of genes was laborious and low throughput.

In 1998 a dramatic technical development was achieved through the identification by Elisabetta Ullu's laboratory of an active RNA interference pathway in *T. brucei*, essentially contemporaneously with its discovery in other systems [168]. Initially discovered through the cytoskeletal perturbation generated (‘FAT’ phenotype) after the expression of a double stranded RNA segment derived from the tubulin gene locus, the ability to effectively silence gene expression in trypanosomes by the expression of double stranded RNA derived from a target gene of interest (generated either as two complementary transcripts from opposing promoters [169,170] or as a single ‘stem loop’ transcript [171,172]) has revolutionised gene function analysis. In a single round of transfection into a cell line already expressing the tetracycline repressor, genes could be silenced or not by the inclusion or absence of tetracycline in culture medium, or doxycycline in the water of infected rodents. Importantly, the organelle and cell structural markers characterised earlier provided the simple phenotypic assays to monitor effects on cell shape and cell cycle progression, providing rapid visual clues to the function of silenced genes. Since its discovery, over 500 papers have described the use of RNAi to analyse gene function in trypanosomes [173], reflecting the rapid application of this important tool in trypanosome cell molecular biology, this being assisted by many improvements and refinements in the sophistication of constructs used for transfection and the increasing availability of selectable markers.

As well as using RNAi for reverse genetic analysis of gene function, it is increasingly used as a tool for forward genetic selection. The first genome wide RNAi library was created in Paul Englund's lab, which applied concanavalin A binding selection to isolate parasites unable to produce EP procyclin [174]. This identified hexokinase silencing as an unexpected regulator of procyclin expression but was laborious, requiring 5 × 10^9^ parasites to be transfected in 50 transfections to provide sufficient genome coverage.

For bloodstream forms, the lower transfection efficiency precluded forward genetic approaches until higher efficiencies were achieved through the use of Amaxa nucleofector technology [175]. Integration efficiency was also improved by the creation of targeted double stranded breaks using a meganuclease that acts as a catalyst for homologous recombination at a specific target site [176–178]. Through these approaches genome-wide phenotypic approaches became feasible in the disease-relevant bloodstream form of the parasite, selections first being applied to drug resistance mechanisms. After exposure of RNAi libraries to different therapeutically important drugs, genes whose silencing allowed survival could be identified by isolation of the RNAi inserts enriched in the selected populations by PCR amplification. This resulted in the simultaneous confirmation of the AAT transporters as a resistance target for eflornithine in the Horn and Roditi labs [179,180], with this being followed up by high throughput analysis of selected inserts by RIT-Seq analysis [181]. Application of this approach to identify essential genes [181], genes linked to drug resistance [182], and subsequently to genes associated with quorum sensing pathways in the parasite [107], have provided detailed insight into important phenomena in the parasite unbiased by prior knowledge. This represents an enormously powerful benefit of this approach, which is able to associate genes of no known function with selectable phenotypes, providing a genome-wide annotation of hypothetic protein classes of otherwise unpredictable function.

## 9 Drug discovery

In the last 25 years there has been an explosion in research directly focused on therapies for African trypanosomiasis, with 21% of manuscripts in 1990 mentioning ‘drug + brucei’ *vs.* ‘brucei’ alone (89/418 papers) whilst in 2013, this had increased to 41% (275/666 papers). This effort has not been accompanied by the emergence of many new drugs to treat the disease, but very promising progress is being made (Fig. 1B). In 1990, eflornithine was first licensed for use against *T. b. gambiense*, and since then NECT (nifurtimox eflornithine combination therapy; launched in 2009) has also been applied successfully. This reduces the time needed for treatment from 14 to 10 days and the number of intravenous doses required is reduced by 75%, with an overall halving of the therapeutic cost [183]. Major effort has also been put in to the development of inhibitors for new targets identified as essential to the parasite after experimental validation, genome prediction of pathway dependencies or from cell based high throughput screening of drug libraries against parasites in culture. Target based approaches have identified N-myristoyl transferase as potentially suitable for therapeutic inhibition [184] but for other targets molecular or *in vitro* inhibition assays have not always proven reliable indicators of *in vivo* efficacy [185]. In consequence there has been some shift in focus away from molecular targets defined *a priori* towards high throughput phenotypic screening to rapidly identify cell permeable inhibitors that are toxic to the parasite but not host cells. This has been enabled by the development of robotic screening approaches and partnerships between academia and pharmaceutical companies allowing access to expertise, equipment and compound libraries not otherwise available or practicable in a university setting. Promising outcomes from this phenotypic screening approach have been development of the orally effective drugs fexinidazole, which is currently in phase III clinical trials and oxaborole SCYX-7158 in phase I clinical trials [186].

These efforts provide encouragement that effective new therapies will be delivered for human African trypanosomiasis, but major challenges to disease control remain. Firstly, drug development is enormously expensive yet trypanosomiasis therapy offers no prospect of a commercial return. This places the emphasis of drug development on governments, charities and industrial philanthropy, when other diseases demand greater attention or are more immediately pressing. This challenge has been met by the establishment of effect public–private partnerships, for example DNDi, bringing partners together to establish collaborations and accelerate drug development. Secondly, any developed therapies may be beyond the health budgets of afflicted regions, particularly those facing ongoing political and economic upheaval. Thirdly, due to a decrease in the number of cases of human African trypanosomiasis in recent years the impact of trypanosomiasis on humans is now largely indirect, through the effects of the parasite on livestock. However, the major livestock trypanosomatids, *T. congolense* and *T. vivax*, have not been the focus of most drug screening efforts and their biology and biochemistry may be sufficiently different to restrict drug efficacy, a point well illustrated by the relative insensitivity of *T. b. rhodesiense* to eflornithine when compared to *T. b. gambiense* [187]. Moreover, the mode of action of drugs used against the livestock parasites is relatively unexplored with even ethidium bromide (a commonly used trypanocide in livestock) only being recently characterised[188]. Finally, the importance of trypanosomiasis as a zoonosis is often overlooked, such that preventing human infection requires animal infections to be specifically and actively targeted [50], a major challenge given the broad host range of human infecting trypanosomes.

Despite these challenges, molecular parasitology has offered direct opportunities for control, an example being the exploitation of the mechanism of human resistance to animal trypanosomes through the engineering of cattle expressing APOL1 [189]. Conversely, drug screening efforts have the potential to provide new biological insight, through the identification of tool compounds that assist the dissection of biological processes particularly when in combination with genome wide approaches to identify molecular pathways perturbed by particular inhibitors [190]. Hence, there is every prospect that brute force drug screening efforts will reap rewards not only in new therapies, but also through illuminating areas of parasite biology that would otherwise not be predicted through conventional hypothesis-driven research.

## 10 Future perspectives

The last 25 years of research into African trypanosomes has uncovered many key aspects of the parasite's biology. During this time, the number of human cases of the disease have increased and then decreased again, such that current reported cases are less than 10,000 per year. Although this has raised hopes for the elimination of the disease, it is cautionary to note that the level of infections currently are still higher than in the 1960s and that the main foci of human infections, the Democratic Republic of Congo, Central African Republic and South Sudan are currently undergoing violent political unrest, conditions that are ideal for resurgence of trypanosomiasis in the coming years. Furthermore, the livestock disease continues to have major impact throughout sub-Saharan Africa, and resistance is an increasing problem for farmers, not least due to the unregulated drug supply chain.

Trypanosomes have also maintained their position as fascinating biological models and, through their evolutionarily divergent position, have continued to reveal novel phenomena that inform all of eukaryotic biology. Recent examples include the unusual epigenetic mechanisms that trypanosomes exhibit [124], their novel mechanisms of chromosome segregation [79] and flagellar biology. This novelty is important and maintains trypanosomes as an interesting system for general molecular and cell biologists, particularly given the range of tools available now to study and manipulate these parasites.

Indeed, now that the genome sequence has been determined, many obviously conserved processes have been dissected, and the core molecular cartography of mRNA and protein expression has provided a framework for more hypothesis-driven analyses, the next 25 years promise exciting new discoveries focussed on the distinct biology of the parasite and the abundance of genes for which no function can be predicted *a priori*. The future will be unexpected, but some key areas need to be explored further. For example, trypanosome biologists, with notable exceptions, have tended to ignore the interactions of the parasite with the host immune system, except at the most rudimentary level. It is likely that the sophistication of these parasites, able to sustain chronic infections in multiple hosts, will reveal new immunomodulatory phenomena of importance to the infection dynamics of the parasite. This is also true in the tsetse fly vector, where immune interactions with arthropod defences will have strong impact on the transmission capability of trypanosomes between hosts. Also the interactions of different parasites populations in the field remain poorly characterised. The *Plasmodium* field has been particularly effective at analysing and studying field strains to understand the biology of that parasite, but in trypanosomes population analyses in a field setting have been relatively limited [191–193], with a strong focus on only a single laboratory adapted strain whose biology and even drug sensitivity may show important differences. The interaction of *T. brucei* with other trypanosomes (and potentially other parasites) is also an emerging theme – with social interactions between trypanosomes both in the tsetse fly (social motility) [194,195] and in the bloodstream (quorum sensing) [196] providing an important component to their life history with fascinating evolutionary and cell biological implications, relevant across a broad range of microbes.

As well as these whole organism questions, reductionist details are still very incomplete in many areas of trypanosome molecular biology. Despite the characterisation of many molecular components, we still do not understand how gene regulation is co-ordinated, or how signalling pathways connect to generate a coherent cellular response to the environment. Even the machinery underlying VSG expression control and switching is only characterised in outline, as are the molecular controls underlying the parasite's cell cycle and the co-ordination between the nuclear and organelle replication and division cycles. The number of manuscripts published on *Trypanosoma brucei* has increased every year and shows no sign of diminishing. It is incumbent on the field to continue to ask interesting questions and discover striking novelty, rather than just increasing detail on established processes, in order to retain the interest of the wider scientific community and provide important new insight into basic biological processes. This is the challenge for the next 25 years.
